# DFENet: A Novel Dual-Path Feature Extraction Network for Semantic Segmentation of Remote Sensing Images

**DOI:** 10.3390/jimaging12030141

**Published:** 2026-03-23

**Authors:** Li Cao, Zishang Liu, Yan Wang, Run Gao

**Affiliations:** School of Electrical and Electronic Engineering, Wuhan Polytechnic University, Wuhan 430023, China; 12591@whpu.edu.cn (L.C.); w2268388154@163.com (Y.W.); 136360597551@163.com (R.G.)

**Keywords:** remote sensing images, dual-path architecture, discrete wavelet transform, frequency-domain information

## Abstract

Semantic segmentation of remote sensing images (RSIs) is a fundamental task in geoscience research. However, designing efficient feature fusion modules remains challenging for existing dual-branch or multi-branch architectures. Furthermore, existing deep learning-based architectures predominantly concentrate on spatial feature modeling and context capturing while inherently neglecting the exploration and utilization of critical frequency-domain features, which is crucial for addressing issues of semantic confusion and blurred boundaries in complex remote sensing scenes. To address the challenges of feature fusion and the lack of frequency-domain information, we propose a novel dual-path feature extraction network (DFENet) in this paper. Specifically, a dual-path module (DPM) is developed in DFENet to extract global and local features, respectively. In the global path, after applying the channel splitting strategy, four feature extraction strategies are innovatively integrated to extract global features from different granularities. According to the strategy of supplementing frequency-domain information, a frequency-domain feature extraction block (FFEB) dominated by discrete Wavelet transform (DWT) is designed to effectively captures both high- and low-frequency components. Experimental results show that our method outperforms existing state-of-the-art methods in terms of segmentation performance, achieving a mean intersection over union (mIoU) of 83.09% on the ISPRS Vaihingen dataset and 86.05% on the ISPRS Potsdam dataset.

## 1. Introduction

Semantic segmentation of remote sensing images is defined as the classification of pixels within large-scale remote sensing imagery. The purpose of this process is to enhance the analysis and interpretation of remote sensing data, thereby aiding researchers in gaining deeper insights into actual conditions of the earth’s surface. The utilization of automated analysis and interpretation methodologies provides substantial support for a variety of downstream tasks and applications, including land cover mapping, environmental monitoring, and disaster management. However, the semantic segmentation process of remote sensing images faces many unique challenges. In high-spatial-resolution (HSR) images, objects vary significantly in scale; thus, rendering boundary precision becomes particularly critical. Furthermore, remote sensing images contain not only objects of interest, such as buildings and bridges, but also background elements, including water bodies and roads, all of which require accurate segmentation. The proportion of foreground objects in these images are typically negligible in comparison to the background, thereby creating an imbalance that causes models to favor background features during training. This, in turn, has a detrimental effect on the quality of foreground segmentation. Consequently, a sophisticated approach is imperative to address these challenges.

In recent years, deep learning-based methods have significantly improved the accuracy and efficiency of semantic segmentation. Among them, CNN-based models [1,2] excel in capturing local information through convolutional operations. Fully convolutional networks (FCN) [3] introduced per-pixel classification, followed by U-net [4] with a symmetric encoder–decoder structure. PSPNet [5]. FPN [6] expanded receptive fields with spatial pyramid pooling, while other methods [7], such as orientation attention network [8] and stair fusion network [9], incorporated attention mechanisms. Although these CNN-based methods achieve strong local representation capability, their convolutional nature inherently restricts the modeling of long-range contextual dependencies. Transformer-based models [10,11,12] leverage self-attention mechanisms [13] to model long-range dependencies. The boundary-aware multiscale network [14] introduced a scale attention module to construct long-range dependencies. Mixed-mask Transformer [15] used a hierarchical encoder for multi-scale learning. However, the quadratic computational complexity of self-attention leads to high computational and memory costs when processing high-resolution remote sensing images. Despite these methods improving the accuracy of semantic segmentation in remote sensing images, limitations still exist. The former fails to effectively capture global contextual information due to its limited receptive fields, while the latter faces substantial computational challenges when processing high-resolution and large-scale remote sensing data.

Recently, Mamba [16], based on State Space Models (SSM) [17], has emerged as an alternative capable of modeling long-range dependencies while maintaining linear computational complexity. Subsequently, it has been applied to remote sensing image semantic segmentation tasks. Pan-Mamba [18] incorporates channel-swapped Mamba and cross-mode Mamba for panchromatic sharpening tasks. RSMamba [19], on the other hand, utilizes multi-path VSS blocks suitable for large-scale image interpretation. Both of them directly adopt VSS blocks to replace the corresponding modules in existing networks. However, due to the complexity of ground object scenes in remote sensing images, relying solely on a single structural model makes it difficult to achieve high segmentation accuracy. To address this challenge, researchers have proposed various solutions, such as multi-scale feature analysis and multi-branch structures for feature extraction. Among them, multi-scale feature analysis methods [20,21,22] have included multi-level feature pyramid networks (FPN). By conducting detailed analysis of object representations at different scales, they aim to improve recognition accuracy and adaptability. RS3Mamba [23] employed a dual-branch structure to extract global and local information, then it supplemented information via a fusion module, thereby improving the accuracy of semantic segmentation of remote sensing images.

Although the aforementioned methods achieve good segmentation results, they typically rely solely on spatial-domain features while overlooking the unique value of frequency-domain information. Frequency-domain analysis is more sensitive to features such as grayscale gradients and textures, which are crucial for addressing issues of semantic confusion and blurred boundaries in remote sensing scenes caused by complex lighting conditions or ambiguous land cover boundaries. Thus, introducing frequency-domain features becomes a core direction for breaking through the current bottleneck in segmentation accuracy.

**Figure 1:**
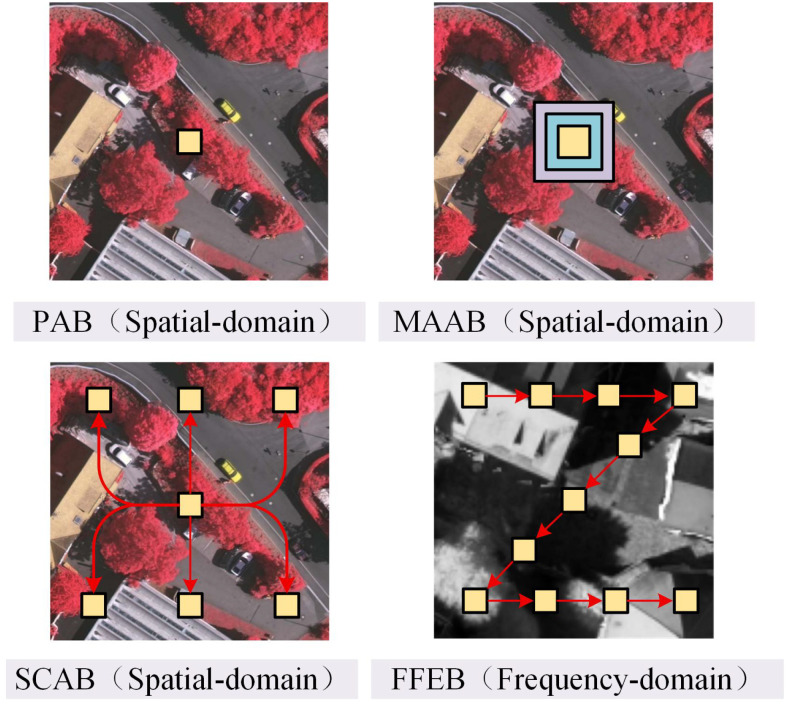
Schematic diagram of the ‘from point to surface’ principle.

Inspired by this, we propose a novel dual-path feature extraction network (DFENet) incorporating frequency-domain information in this paper. Based on the traditional encoder–decoder architecture, in each encoding stage, DFENet mainly consists of a conventional path for local feature extraction, a newly designed path that integrates multiple strategies for global feature extraction, and a feature enhancement module (FEM) used to further enhance the features of the two paths before inputting them to the current decoding stage. Specifically, the global path is developed to first employ a channel splitting strategy, and then, based on the principle of ‘from point to surface’ [24] shown in Figure 1, integrate the point attention block (PAB), the multi-scale attention aggregation block (MAAB), the spatial context aware block (SCAB) and the frequency-domain feature extraction block (FFEB) to extract global features from diverse granularities.

Notably, the FFEB is designed to make up for the deficiency of Wavelet transform-based methods that only deal with easy-to-handle low-frequency information and ignore the equally important high-frequency information [25]. The contributions of this study can be summarized as follows:

(1) A novel dual-path feature extraction network (DFENet) is proposed for semantic segmentation of remote sensing images. In each stage, a dual-path module (DPM) is designed to independently extract global and detailed features, thereby achieving an improvement in segmentation accuracy of remote sensing images.

(2) A global path is developed, which contains multiple feature extraction strategies from different granularities to provide rich hierarchy and complete global information, thereby enhancing the model’s robustness of semantic segmentation in complex remote sensing scenes.

(3) The FFEB is designed to effectively supplement the frequency-domain information, which is often overlooked during feature extraction, with the aim of further increasing the segmentation performance by about 2%.

(4) Extensive experiments are conducted on two public datasets to verify the effectiveness and superiority of our proposed DFENet in terms of segmentation performance. Specifically, DFENet attains 91.55% overall accuracy (OA), 90.41% mean F1-score (mF1), and 83.09% mean intersection over union (mIoU) on the ISPRS Vaihingen dataset, while achieving 91.08% OA, 92.21% mF1, and 85.89% mIoU on the ISPRS Potsdam dataset.

## 2. Related Work

In this section, we review existing spatial-domain methods for remote sensing image segmentation and analyze the advantages of frequency-domain approaches for image segmentation. Moreover, we emphasize the importance of incorporating frequency-domain information for accurate segmentation of high-resolution remote sensing images in urban environments.

### 2.1. Methods Based on Spatial-Domain

With the rapid advancement of deep learning, CNN-based approaches have become the mainstream in remote sensing image segmentation. Since the introduction of a fully convolutional network [3], convolutional architectures have achieved remarkable success in semantic segmentation of remote sensing images owing to their strong capability for local feature extraction. Among them, U-Net [4] pioneered the encoder–decoder framework, where the encoder extracts hierarchical features through multiple convolutional and pooling layers, and the decoder progressively restores spatial resolution via deconvolution and upsampling operations. Moreover, skip connections enable feature fusion between encoder and decoder sections, effectively alleviating information loss and improving segmentation accuracy. These CNN-based methods exhibit high computational efficiency and robustness against noise and occlusion. However, their limited receptive field constrains the ability to capture global semantic context. Consequently, their performance in complex urban scenes characterized by fine textures, small-scale structures, and varying illumination remains suboptimal, with restricted generalization capability.

To overcome the limitations of CNNs in global feature modeling, Transformer-based architectures have been introduced. By leveraging self-attention mechanisms, Transformer effectively captured long-range dependencies, thereby enhancing global feature representation [26]. For instance, the Swin Transformer [27] employed a hierarchical sliding-window designed to balance local and global feature extraction, achieving superior performance in small-object detection tasks. Additionally, its spatial attention interleaved cascade (SAIEC) architecture further improved segmentation accuracy. Nevertheless, Transformer-based models require pairwise correlation computations across all spatial positions, leading to substantial computational overhead and prolonged training and inference time. Although subsequent optimizations [28] have alleviated these issues to some extent, they still face efficiency bottlenecks when processing high-resolution remote sensing images.

Recently, SSMs have emerged as promising alternatives to attention mechanisms, demonstrating strong capability in capturing long-range dependencies with reduced computational complexity. Mamba, a linear-time SSM, achieved efficient global modeling through selective state updates. Building upon this foundation, Vision Mamba [29] and VMamba [30] introduced bidirectional or cross-scan mechanisms to transform two-dimensional spatial features into sequential representations, making them more suitable for visual tasks. As research has advanced, Mamba-like architectures have been applied to remote sensing tasks, achieving a favorable balance between accuracy and efficiency. However, most existing spatial-domain methods overlook frequency-domain information, which is crucial for accurately representing complex textures, blurred edges, and shadowed regions in high-resolution images [31,32]. As frequency-domain information provides complementary fine-grained semantic cues, combining it with spatial-domain information is a viable approach to enhance the performance of existing semantic segmentation methods for remote sensing images.

### 2.2. Methods Based on Frequency-Domain

In recent years, wavelet transforms have been widely applied in image segmentation owing to their powerful capabilities for multi-resolution analysis. Unlike traditional Fourier transforms, wavelet transforms enable localized analysis on signals in both the spatial and frequency domains, effectively capturing detailed and global image features simultaneously. This property provides distinct advantages for processing remote sensing images that exhibit complex textures, abundant edge information, and multi-scale structures. Previous studies have demonstrated that performing wavelet decomposition prior to segmentation can separate image components across different frequency bands, thereby enhancing target–background contrast and improving segmentation accuracy [33,34].

Wavelet-based segmentation methods can generally be categorized into wavelet thresholding, wavelet-domain clustering, and multi-scale fusion approaches integrated with deep learning architectures. Among them, classical wavelet thresholding effectively suppresses noise while preserving essential edge structures, whereas multi-scale wavelet segmentation methods incorporating clustering algorithms [35,36] further improved segmentation robustness and regional consistency. Recent research has explored embedding wavelet transforms within CNN and Transformer frameworks to enhance feature representation and model frequency-domain characteristics. Such hybrid approaches achieved complementary fusion between spatial- and frequency-domain features, leading to superior segmentation performance in complex remote sensing scenarios [27,31]. Although wavelet transform has demonstrated significant advantages in image segmentation, it still exhibits clear limitations in the synergistic integration of spatial and frequency-domain features. In particular, existing wavelet-based segmentation methods [22] exhibited restricted utilization of frequency-domain information during the feature extraction phase. Most of these approaches primarily emphasize low-frequency information, which is computationally efficient and effectively captures the global structural characteristics of images, while neglecting high-frequency information. However, high-frequency information plays a crucial role in representing fine-grained details such as edges and textures, which are essential for precise segmentation. As a result, the potential of frequency-domain features remains underexploited in current segmentation frameworks.

## 3. Methodology

Section 3.1 provides a detailed description of the framework of our proposed DFENet. Furthermore, Section 3.2 elaborates on the composition of the global path and the multiple feature extraction blocks it contains, especially the FFEB designed to supplement the frequency-domain information.

### 3.1. Framework of DFENet

**Figure 2:**
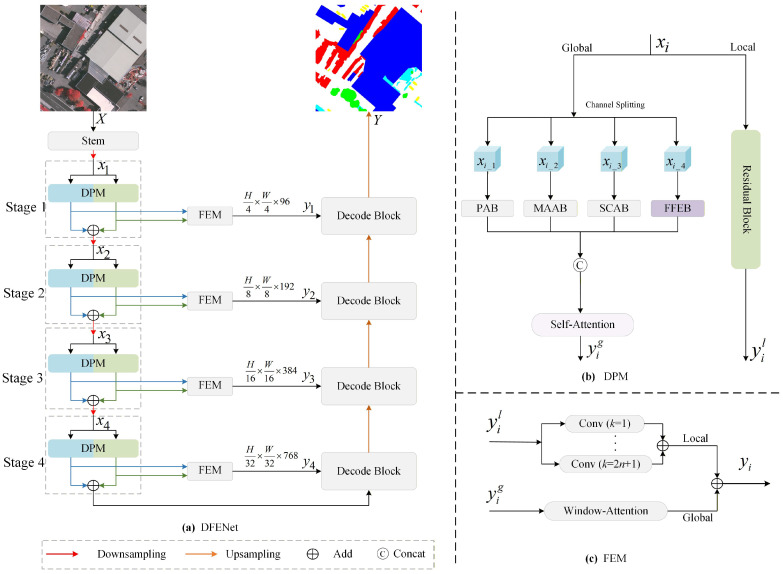
The overall architecture of our proposed DFENet.

The overall architecture of DFENet is illustrated in Figure 2a. Following the traditional multi-level encoder–decoder framework, the network is divided into four hierarchical stages. The encoder progressively reduces the spatial resolution to 1/4, 1/8, 1/16, and 1/32 of the input size through successive downsampling operations. Stages 1 to 4 are configured with output channel dimensions of 96, 192, 384, and 768, respectively. For stage *i*, i∈{1,2,3,4}, the input feature map $x_{i}$ is first processed by a DPM to extract local and global features separately. Subsequently, these two types of features are enhanced and fused in a FEM to generate the stage feature $y_{i}$. Finally, $y_{i}$ is sent to the corresponding decoding stage for subsequent processing.

As shown in Figure 2b, the DPM concurrently incorporates a local path and a global path. The former takes the lightweight ResNet-18 as its backbone and extracts local detailed features $y_{i}^{l}$ through residual convolution operations. The latter first performs the channel splitting strategy on $x_{i}$, then it extracts multi-granularity global features via four different global feature extraction blocks before concatenating them to obtain the global features $y_{i}^{g}$.

The detailed structure of the FEM is illustrated in Figure 2c, which includes a window-attention [37] block and multiple convolutional blocks. Among them, the former enhances the features from the global path by leveraging its long-range modeling capability, while the latter enhances the features from the local path by utilizing convolutions of different sizes. The formula is expressed as follows:

(1) $$y_{i}=\sum_{k=1}^{k=2n+1}{\mathrm{Conv}}_{k\times k}\left(y_{i}^{l}\right)+\mathrm{WA}\left(y_{i}^{g}\right),n\in N$$

where ${\mathrm{Conv}}_{k\times k}\left(\cdot \right)$ denotes convolution with kernel size *k*, and WA(·) denotes window-attention.

In addition, for the decoder section, the UNetformer decoder [38] is employed, which is specifically designed for the semantic segmentation of remote sensing images.

### 3.2. Structural Design of the Global Path

As shown in Figure 2b, the global path adopts a channel splitting strategy. This channel splitting strategy preserves the spatial resolution while maintaining a cooperative balance between multi-scale feature extraction and computational efficiency. At each stage, the total number of channels in the input feature map is evenly divided into four groups along the channel dimension, with each group containing an equal number of channels. Specifically, the input feature map $x_{i}$ is divided into $\left\{x_{i\_1},x_{i\_2},x_{i\_3},x_{i\_4}\right\}$, where $x_{i}\in R^{H\times W\times C}$, $x_{i\_n}\in R^{H\times W\times C/4}$ and n∈{1,2,3,4}. Following the principle of ‘from point to surface’, the sub-feature maps $\left\{x_{i\_1},x_{i\_2},x_{i\_3},x_{i\_4}\right\}$ are processed by the PAB, the MAAB, the SCAB, and the FFEB, respectively. The PAB extracts point-level fine-grained spatial features, the MAAB captures multi-scale spatial structural features, the SCAB models global spatial contextual correlation features and the FFEB extracts frequency-domain high and low-frequency features with texture and edge details. These four feature extraction blocks are complementary across three dimensions including feature granularity, representation dimension and contextual perception range.

#### 3.2.1. The Details of PAB

**Figure 3:**
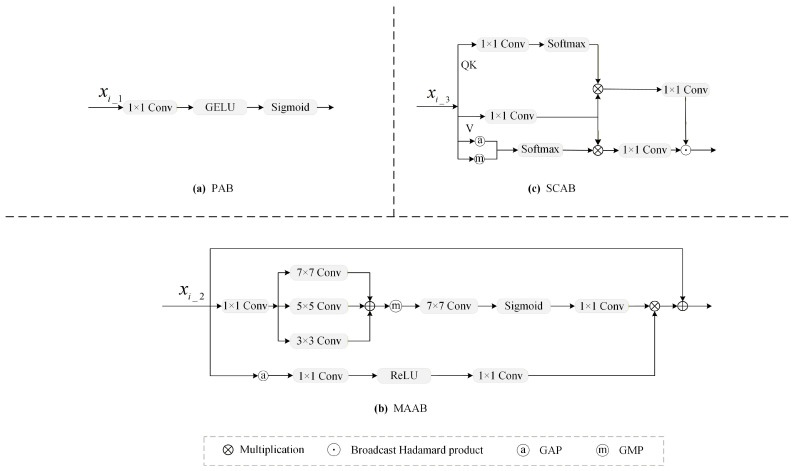
The first three strategies employed for global feature extraction. (**a**) PAB, (**b**) MAAB, (**c**) SCAB.

To extract fine-grained features from $x_{i\_1}$, the PAB [24] first performs a 1 × 1 convolution, then it applies a GELU activation function [39], and finally, it uses a Sigmoid function to generate the attention map $y_{i\_1}^{g}$, as shown in Figure 3a. The formula is expressed as follows:

(2) $$y_{i\_1}^{g}=\sigma \left(\mathrm{GELU}({\mathrm{Conv}}_{1\times 1}\left(x_{i\_1}\right))\right),$$

where σ(·) denotes the Sigmoid function, and GELU(·) denotes the Gaussian error linear unit activation function.

#### 3.2.2. The Details of MAAB

MAAB [40] is used to extract multi-scale features from $x_{i\_2}$, as shown in Figure 3b. Spatial refinement starts with channel projection via a 1 × 1 convolution, reducing the number of channels from *C*/4 to *C*/12. Then, multi-scale fusion is achieved by summing the outputs of convolutions with kernel sizes of 3×3, 5×5, and 7×7. The multi-scale fusion feature $y_{ms}$ is computed as follows:

(3) $$y_{ms}=\sum_{k\in \{1,3,7\}}{\mathrm{Conv}}_{k\times k}\left({\mathrm{Conv}}_{1}\times 1\left(x_{i\_2}\right)\right).$$

Subsequently, spatial features are aggregated using global max pooling (GMP), followed by a 7×7 convolution, a Sigmoid function and a 1 × 1 convolution. The spatial aggregation feature $y_{sa}$ is computed as follows:

(4) $$y_{sa}={\mathrm{Conv}}_{1\times 1}(\sigma \left({\mathrm{Conv}}_{7\times 7}\left(\mathrm{GMP}\left(y_{ms}\right)\right)\right).$$

In parallel, the channel aggregation uses global average pooling (GAP) to reduce dimensions to C/4 × 1 × 1, followed by a 1 × 1 convolution, a ReLU activation function and a 1 × 1 convolution to generate the channel aggregation feature $y_{ca}$:

(5) $$y_{ca}={\mathrm{Conv}}_{1\times 1}\left(\mathrm{ReLU}\left({\mathrm{Conv}}_{1\times 1}\left(\mathrm{GAP}\left(x_{i\_2}\right)\right)\right)\right).$$

Finally, $y_{sa}$ and $y_{ca}$ are fused via an element-wise mutiplication. The output of MAAB $y_{i\_2}^{g}$ is obtained through the following formula:

(6) $$y_{i\_2}^{g}=y_{sa}\cdot \;y_{ca}+x_{i\_2}.$$

MAAB enhances spatial and channel-wise features for subsequent network layers.

#### 3.2.3. The Details of SCAB

SCAB [41] is used to capture spatial relationships among pixels from $x_{i\_3}$. As illustrated in Figure 3c, SCAB integrates three types of information. The first type uses a 1×1 convolution to reduce the dimensions of queries and keys. The second type applies a 1×1 convolution to compute a linear transformation of the feature map. The third type aggregates global contextual information using GAP and GMP. Subsequently, the outputs of the first and third types are multiplied with the second type via matrix multiplication, producing two feature maps that represent cross-channel and cross-spatial contextual information, respectively. These feature maps are further enhanced through a 1×1 convolution to obtain fine-grained features. Finally, the output of SCAM is obtained using the broadcast Hadamard product on these two fine-grained feature maps. The formulas are expressed as follows:

(7) $$y_{T1}=\mathrm{Softmax}\left({\mathrm{Conv}}_{1\times 1}\left(x_{i\_3}\right)\right),$$

(8) $$y_{T2}={\mathrm{Conv}}_{1\times 1}\left(x_{i\_3}\right),$$

(9) $$y_{T3}=\mathrm{Softmax}\left(\mathrm{Cat}\left(\mathrm{GAP}\left(x_{i\_3}\right),\mathrm{GMP}\left(x_{i\_3}\right)\right)\right),$$

(10) $$y_{i\_3}^{g}={\mathrm{Conv}}_{1\times 1}\left(y_{T1}\cdot y_{T2}\right)⊙{\mathrm{Conv}}_{1\times 1}\left(y_{T2}\cdot y_{T3}\right),$$

where ⊙ denotes the Hadamard product (element-wise multiplication), and Cat(·) denotes the feature concatenation operation.

#### 3.2.4. The Details of FFEB

**Figure 4:**
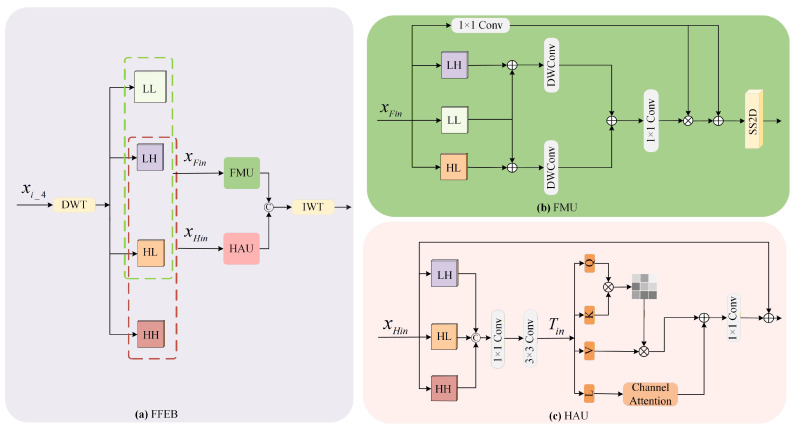
The fourth strategy employed for global feature extraction. (**a**) FFEB, (**b**) frequency Mamba unit (FMU), (**c**) high-frequency attention unit (HAU).

**Figure 5:**
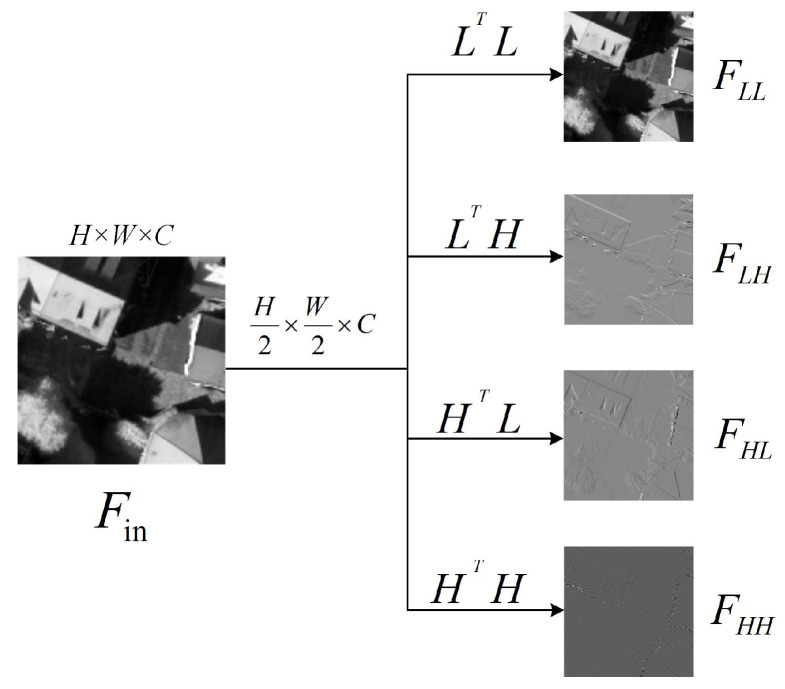
Visualization of wavelet decomposition applied to a feature map.

As shown in Figure 4a, FFEB is designed to supplement the frequency-domain features of $x_{i\_4}$. This block utilizes discrete wavelet transform (DWT) to convert $x_{i\_4}$ into its frequency-domain representations. As shown in Figure 5, DWT employs a low-pass filter $L=[1/\sqrt{2},1/\sqrt{2}]$ and a high-pass filter $H=[-1/\sqrt{2},1/\sqrt{2}]$ to construct four convolution kernels with a stride of 2. These kernels are denoted as ${\mathrm{LL}}^{T}$, ${\mathrm{LH}}^{T}$, ${\mathrm{HL}}^{T}$ and ${\mathrm{HH}}^{T}$. They decompose $x_{i\_4}$ into four wavelet sub-bands. These sub-bands are $F_{\mathrm{LL}}\in R^{H/2\times W/2\times C}$, $F_{LH}\in R^{H/2\times W/2\times C}$, $F_{HL}\in R^{H/2\times W/2\times C}$ and $F_{HH}\in R^{H/2\times W/2\times C}$. Among these sub-bands, $F_{LL}$ preserves the coarse structure of the image, while $F_{LH}$, $F_{HL}$ and $F_{HH}$ contain directional edge information and texture details, respectively.

Unlike conventional wavelet-based methods, considering that $F_{LH}$ and $F_{HL}$ contain not only details such as edges and textures but also certain global contextual information, to comprehensively extract low-frequency features, a frequency Mamba unit (FMU) is designed to process $F_{LL}$, $F_{LH}$ and $F_{HL}$, with the aim of preserving abundant global information. Similarly, to fully capture the high-frequency features, a high-frequency attention unit (HAU) [42] is introduced to process $F_{LH}$, $F_{HL}$, and $F_{HH}$.

The design of the FMU is shown in Figure 4b. To generate the attention map, $F_{LL}$ and $F_{LH}$, as well as $F_{LL}$ and $F_{HL}$, are combined via element-wise addition. After each sum passes through a 3 × 3 depthwise convolution layer, their results are combined again via element-wise addition. The comprehensive global features $F_{cg}$ are computed as follows:

(11) $$F_{\mathrm{cg}}={\mathrm{Conv}}_{1\times 1}\left({\mathrm{DWConv}}_{3\times 3}\left(F_{\mathrm{LL}}+F_{\mathrm{LH}}\right)+{\mathrm{DWConv}}_{3\times 3}\left(F_{LL}+F_{HL}\right)\right).$$

Subsequently, the SS2D mechanism of Mamba is utilized to effectively capture long-range dependencies, and finally, the attention map is generated via the Sigmoid function. This operation can be expressed as follows:

(12) $$y_{Fout}=\mathrm{SS}2D\left(F_{cg}\cdot {\mathrm{Conv}}_{1\times 1}\left(x_{Fin}\right)+{\mathrm{Conv}}_{1\times 1}\left(x_{Fin}\right)\right)$$

where SS2D(·) denotes the SS2D mechanism of Mamba.

For high-frequency $F_{LH}$, $F_{HL}$, and $F_{HH}$, we introduce the HAU. As shown in Figure 4c, given $F_{LH},F_{HL},F_{HH}\in R^{H/2\times W/2\times C/4}$. First, $F_{LH}$, $F_{HL}$, and $F_{HH}$ are concatenated along the channel dimension. The concatenated feature then undergoes a 1×1 convolution followed by a 3×3 convolution to obtain the embedded feature $T_{in}\in R^{4\times H/2\times W/2\times 3C/4}$. The embedded feature $T_{\mathrm{in}}$ is computed as follows:

(13) $$T_{in}={\mathrm{Conv}}_{3\times 3}({\mathrm{Conv}}_{1\times 1}\left(\mathrm{Cat}(F_{LH},F_{HL},F_{HH}))\right).$$

Then, $T_{in}$ is split into four branches, denoted as *Q*, *K*, *V*, and *L*, respectively. Among them, *K* and *V* perform element-wise multiplication operations, *Q* participates in interactive computations, and *L* interacts with a channel attention mechanism. The processed features are then fused, followed by a 1×1 convolution, and finally added back to the original path via a residual connection to output the enhanced feature. The formula is expressed as follows:

(14) $$\left[Q,K,V,L\right]=\mathrm{Split}\left(T_{in}\right),$$

(15) $$y_{Hout}={\mathrm{Conv}}_{1\times 1}\left(\mathrm{Softmax}\left(\frac{QK^{T}}{\sqrt{d_{k}}}\right)V+\mathrm{CA}\left(L\right)\right)+\mathrm{LN}\left(x_{Hin}\right)$$

where LN(·) denotes the layer normalization operation, and CA(·) denotes the channel attention mechanism.

The final output of FFEB $y_{i\_4}^{g}$ is obtained by concatenating $y_{Fout}$ and $y_{Hout}$, and then performing an inverse wavelet transformation (IWT).

## 4. Experiment

### 4.1. Datasets

#### 4.1.1. ISPRS Vaihingen

The ISPRS Vaihingen dataset comprises 16 very high-resolution true orthophotos, each with an average pixel size of 2500 × 2000. Each orthophoto has 3 channels, corresponding to the near-infrared, red, and green channels, respectively. The dataset covers 5 foreground categories, namely *impervious surface*, *building*, *low vegetation*, *tree*, and *car*, along with a background class (i.e., *clutter*). In our experiments, these 16 orthophotos are split into two parts: a training set consisting of 12 patches and a test set containing 4 patches. The training set includes orthophotos with indices 1, 3, 23, 26, 7, 11, 13, 28, 17, 32, 34, and 37, while the test set comprises orthophotos with indices 5, 21, 15, and 30.

#### 4.1.2. ISPRS Potsdam

The ISPRS Potsdam dataset is composed of 24 very high-resolution true orthophotos, each with a pixel size of 6000 × 6000, containing the same category information as the Vaihingen dataset. However, unlike the Vaihingen dataset, the Potsdam dataset has 4 multi-spectral channels, namely near-infrared, red, green, and blue. In our experiments, these 24 orthophotos are divided into two parts: a training set consisting of 18 patches and a test set composed of the remaining 6 patches. The training set includes orthophotos with indices 6_10, 7_10, 2_12, 3_11, 2_10, 7_8, 5_10, 3_12, 5_12, 7_11, 7_9, 6_9, 7_7, 4_12, 6_8, 6_12, 6_7, and 4_11, while the test set comprises orthophotos with indices 2_11, 3_10, 4_10, 5_11, 6_11, and 7_12.

### 4.2. Experimental Setup

All experiments are conducted using the PyTorch v2.0.0 deep learning framework on an NVIDIA A40 GPU with 48 GB of memory. All the models are trained using the Stochastic Gradient Descent (SGD) optimizer for 50 epochs, with momentum and weight decay set to 0.9 and 0.0005, respectively. The initial learning rate is set to 0.01 and is subsequently adjusted following the cosine annealing schedule. During training, the batch size is maintained at 24.

To achieve efficient learning, each training orthophoto is randomly cropped into 256 × 256 patches. Meanwhile, a variety of data augmentation techniques are applied to enhance the generalization ability of the model, specifically including random horizontal flipping, random vertical flipping, and random rotation at different angles.

In order to evaluate the segmentation performance, three widely recognized metrics in the field of semantic segmentation are selected: OA, mF1, and mIoU. Based on the cumulative confusion matrix, the calculations of OA, mF1, and mIoU are expressed as follows:

(16) $$\mathrm{OA}=\frac{\sum_{k=1}^{N}TP_{k}+TN_{k}}{\sum_{k=1}^{N}TP_{k}+FP_{k}+TN_{k}+FN_{k}},$$

(17) $$Q_{p}=\frac{1}{N}\sum_{k=1}^{N}\frac{TP_{k}}{TP_{k}+FP_{k}},$$

(18) $$Q_{r}=\frac{1}{N}\sum_{k=1}^{N}\frac{TP_{k}}{TP_{k}+FN_{k}},$$

(19) $$F1=2\times \frac{Q_{p}\times Q_{r}}{Q_{p}+Q_{r}},$$

(20) $$\mathrm{mIoU}=\frac{1}{N}\sum_{k=1}^{N}\frac{TP_{k}}{TP_{k}+FP_{k}+FN_{k}},$$

where ${\mathrm{TP}}_{k}$, ${\mathrm{FP}}_{k}$, ${\mathrm{TN}}_{k}$, and ${\mathrm{FN}}_{k}$ denote true positives, false positives, true negatives, and false negatives, respectively, for objects indexed as class *k*. Specifically, we incorporated *Clutter* into the evaluation of OA and calculated the mF1 and mIoU values for five foreground classes.

In addition, we also use two evaluation metrics, namely Floating Point Operations (FLOPs) and the number of model parameters, to assess the complexity of the models. FLOPs characterize the computational complexity of the network, and the number of parameters reflects the scale and structural complexity of the model. Ideally, an effective model should have high segmentation performance while maintaining a low model complexity.

### 4.3. Performance Comparison

We compare the proposed DFENet with nine state-of-the-art methods, including three CNN-based models, SFFNet [43], UNetFormer [38], and MAResU-Net [44]; two Transformer-based models, TransUNet [45] and CMTFNet [46]; two Mamba-based models, RS3Mamba [23] and UNetMamba [47]; and two frequency modeling-based models, DECS-Net [48] and MIFNet [31].

(1) *Comparison on the Vaihingen Dataset*: As shown in Table 1, DFENet achieves state-of-the-art performance, attaining the highest scores in OA (91.55%), mF1 (90.41%), and mIoU (83.09%). Our approach demonstrates signiffcant improvements over key baseline models, outperforming RS3Mamba by increments of 0.25% (91.30% vs. 91.55%) in OA; 0.21% (90.20% vs. 90.41%) in mF1; and 0.56% (82.56% vs. 83.09%) in mIoU. Notably, compared with SFFNet, which also adopts spatial–frequency processing, DFENet achieves substantial improvements by increments of 0.76% (90.79% vs. 91.55%) in OA, 5.96% (84.45% vs. 90.41%) in mF1, and 9.24% (73.85% vs. 83.09%) in mIoU. Compared with the latest frequency-domain model, although DECSNet achieves the optimal segmentation performance on the *building* category, our model outperforms DECSNet on all other categories. Even though our model does not attain the best F1-score on *impervious surface* and *building*, the gaps from the optimal results are only 0.01% and 0.12%, respectively. Furthermore, in challenging categories such as *low vegetation*, *car*, and *tree*, DFENet yields higher F1 and IoU values than the other compared models.

**Figure 6:**
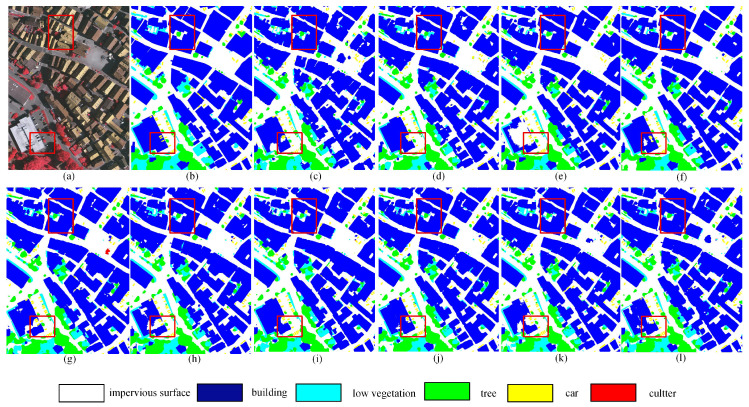
Qualitative comparisons on the ISPRS Vaihingen dataset. (**a**) NIRRG image, (**b**) Ground truth, (**c**) SFFNet, (**d**) UNetMamba, (**e**) TransUNet, (**f**) UNetFormer, (**g**) MAResU-Net, (**h**) CMTFNet, (**i**) RS3Mamba, (**j**) DECSNet, (**k**) MIFNet, (**l**) Our proposed DFENet.

The qualitative results of DFENet are illustrated in Figure 6j, where two red boxes highlight its capability in capturing complex textures and boundary details. The upper box illustrates shadow occlusion and boundary refinement, while the lower box shows texture discrimination among spectrally similar classes. Our method produces segmentation masks with high fidelity, featuring accurate, smooth, and complete object boundaries compared with the ground truth. In the upper box (*building* and *tree*), DFENet generates continuous *building* contours and accurately segments *tree* within shadowed areas. In contrast, RS3Mamba yields jagged *building* edges, and DECSNet confuses the challenging *low vegetation* with *tree*. Similarly, both UNetMamba and TransUNet suffer from the confusion of two or more categories, and SFFNet not only generates jagged edges but also confuses *buildings* with *low vegetation*, resulting in misclassifications. In the lower box, compared to RS3Mamba, the segmented *buildings* are noticeably more complete. MIFNet produces incomplete buildings and fails to fully segment vehicles occluded by shadows. Although DFENet shows slightly lower completeness in *building* segmentation within the selected region compared with UNetMamba, it substantially outperforms the latter in boundary delineation and *low-vegetation* segmentation. Overall, DFENet exhibits significant improvements in segmentation accuracy and boundary refinement compared with the other methods.

(2) *Comparison on the Potsdam Dataset*: Experimental results on the Potsdam dataset are consistent with those on the Vaihingen benchmark. As shown in Table 2, DFENet achieves state-of-the-art performance, attaining the highest scores in OA (91.08%), mF1 (92.21%), and mIoU (85.89%). It also achieves the highest scores in the *impervious surface* and *building* categories. Compared with RS3Mamba, DFENet improves the performance by increments of 0.31% (90.77% vs. 91.08%) in OA, 0.32% (91.89% vs. 92.21%) in mF1, and 0.65% (85.24% vs. 85.89%) in mIoU. Compared with SFFNet, DFENet achieves larger increments of 3.93% (87.15% vs. 91.08%) in OA, 4.25% (87.96% vs. 92.21%) in mF1, and 6.80% (79.09% vs. 85.89%) in mIoU. Compared with the latest frequency-domain model MIFNet, although MIFNet achieves the optimal segmentation performance on the *car* category, our model outperforms MIFNet on *impervious surface* and *building*, and it achieves 0.07%, 0.14% and 0.08% higher values in OA, mF1 and mIoU, respectively. Notably, the OA obtained on the Potsdam dataset is lower than that on the Vaihingen dataset. This may be attributed to the larger number of small objects and higher background complexity in the Potsdam dataset.

**Figure 7:**
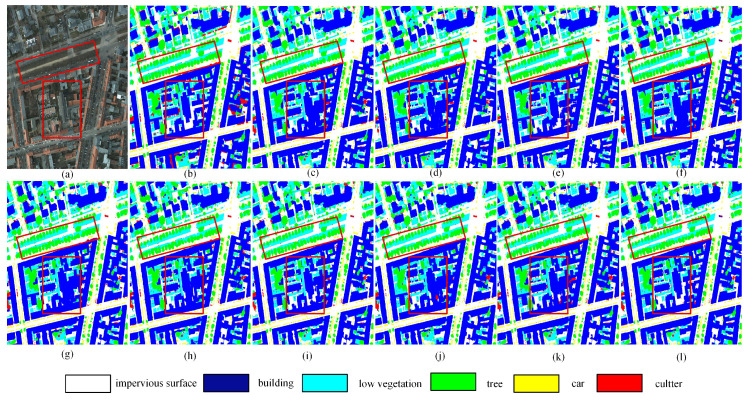
Qualitative comparisons on the ISPRS Potsdam dataset. (**a**) RGB image, (**b**) Ground truth, (**c**) SFFNet, (**d**) UNetMamba, (**e**) TransUNet, (**f**) UNetFormer, (**g**) MAResU-Net, (**h**) CMTFNet, (**i**) RS3Mamba, (**j**) DECSNet, (**k**) MIFNet, (**l**) Our proposed DFENet.

Figure 7j further demonstrates the effectiveness of DFENet, where two red boxes highlight its capability in capturing complex textures and boundary details. The two red boxes clearly illustrate its advantages in capturing complex textures and boundary details. The upper box illustrates DFENet’s accurate category discrimination, particularly for challenging classes such as *trees*, *low vegetation*, and *cars*. DFENet achieves precise separation between *trees* and *low vegetation*, which is closely aligned with the ground truth, while MIFNet fails to maintain precise separation between *low vegetation* and *cars*, and both RS3Mamba and SFFNet exhibit obvious confusion and misclassification artifacts among these categories. The lower box emphasizes boundary integrity and structural consistency, demonstrating that the segmentation masks produced by DFENet are smooth, coherent, and highly consistent with the reference annotations.

Table 3 presents the quantitative comparison between DFENet and state-of-the-art methods. It can be observed that UNetFormer exhibits the lowest complexity, with only 2.94 G FLOPs and 11.69 M parameters, while maintaining an mIoU of 82.44%. In contrast, TransUNet has the largest parameter count of 105.32 M and the highest FLOPs of 38.57 G among all compared methods. Our proposed DFENet yields 16.56 G FLOPs and 95.05 M parameters, which are higher than those of lightweight models such as UNetFormer and UNetMamba, and its parameter count is second only to TransUNet. Despite its relatively higher computational and parametric complexity compared with some lightweight baseline methods, DFENet achieves the highest segmentation performance, with an mIoU of 83.09%.

### 4.4. Analysis and Discussion

Based on the above comparative experiments, it can be concluded that DFENet exhibits significantly stronger generalization ability than lightweight models such as UNetFormer. DFENet can capture multi-granularity spatial and frequency-domain features, thereby achieving better performance in boundary refinement and category discrimination, especially for challenging categories including *low vegetation*, *trees*, and *cars*. Meanwhile, DFENet also has limitations. The dual-path design and channel splitting strategy lead to a moderate increase in the model’s FLOPs and parameters, resulting in higher computational costs than lightweight architectures, which limits its application in resource-constrained deployment scenarios. The moderate increase in model complexity is accompanied by consistently excellent segmentation performance, reflecting a reasonable trade-off between accuracy and resource requirements.

### 4.5. Ablation Study

To evaluate the effectiveness of the proposed DFENet, ablation experiments were conducted on the Vaihingen dataset. As shown in Table 4, when only the local feature extraction path was employed, the model achieved an mIoU of 81.97%, whereas using only the global path resulted in an mIoU of 78.79%. These results indicate that the two paths are complementary, and their joint utilization in the complete DFENet yields the best overall performance, achieving an mIoU of 83.09%.

Furthermore, to assess the contribution of multiple feature extraction strategies used in the developed global path, additional ablation experiments were conducted. As shown in Table 5, removing any one of feature extraction strategies results in a noticeable reduction in mIoU, confirming that the multiple feature extraction strategies based on the principle of ‘from point to surface’ is crucial for maintaining optimal performance. This validates the effectiveness and rationality of the developed global path in enhancing global feature representation.

Additionally, ablation experiments were conducted on the Vaihingen dataset to evaluate the effectiveness of the designed FFEB. As shown in Table 6, when processing low-frequency information using only the FMU, the model achieved an mIoU of 82.41%, whereas using only the HAU resulted in an mIoU of 82.36%. By using both of them, the complete FFEB provides the best performance, achieving an optimal mIoU of 83.09%.

## 5. Conclusions

In this paper, we propose DFENet, a novel dual-path feature extraction network for semantic segmentation of remote sensing images. On the basis on a conventional encoder–decoder architecture, DFENet incorporates a DPM in each encoding stage to capture both local and global representations. In the global path, four different feature extraction blocks based on the principle of ‘from point to surface’ are integrated to extract features at different granularities. Among them, the FFEB designed based on the strategy of supplementing frequency-domain information effectively captures both high- and low-frequency components. Extensive comparisons with state-of-the-art methods demonstrate the superior segmentation performance of DFENet. Experimental results on two representative remote sensing datasets further validate its effectiveness and generalization capability, with a moderate increase in computational cost and model parameters. Future work will focus on exploring more advanced frequency-domain representations and more lightweight models to improve segmentation accuracy and computational efficiency.

## Figures and Tables

**Table 1:** Quantitative comparison results on the Vaihingen dataset. The segmentation accuracy of each category is presented using F1/IoU. The best values are in red.

Method	Impervious Surface	Building	Low Vegetation	Tree	Car	OA (%)	mF1 (%)	mIoU (%)
SFFNet	89.11/80.36	93.82/88.35	75.58/60.74	88.81/79.86	74.93/59.90	90.79	84.45	73.85
UNetMamba	91.82/84.88	96.10/92.49	79.86/66.47	90.77/83.09	85.81/75.13	90.87	88.87	80.42
TransUNet	92.21/85.54	96.10/92.48	80.79/67.77	90.87/83.27	89.60/81.16	91.21	89.91	82.04
UNetFormer	92.23/85.58	96.34/92.93	80.54/67.70	91.04/83.55	90.37/82.43	91.29	90.14	82.44
MAResU-Net	92.66/86.33	96.84/93.87	80.57/67.47	90.84/83.22	89.93/81.71	91.50	90.17	82.51
CMTFNet	92.68/86.37	96.71/93.63	80.47/67.33	90.78/83.11	90.22/82.18	91.42	90.17	82.52
RS3Mamba	92.69/86.38	96.67/93.55	80.54/67.42	90.59/82.79	90.49/82.64	91.30	90.20	82.56
DECSNet	92.56/86.14	96.87/93.92	79.85/66.46	90.85/83.23	88.36/79.15	91.34	89.70	81.79
MIFNet	92.50/86.04	96.78/93.75	80.75/67.72	91.05/83.57	90.38/82.44	91.43	90.29	82.71
DFENet	92.68/86.38	96.75/93.70	80.81/67.70	91.27/83.94	90.95/83.40	91.55	90.41	83.09

**Table 2:** Quantitative comparison results on the Potsdam dataset. The segmentation accuracy of each category is presented using F1/IoU. The best values are in red.

Method	Impervious Surface	Building	Low Vegetation	Tree	Car	OA (%)	mF1 (%)	mIoU (%)
SFFNet	91.61/82.47	96.82/89.75	83.58/69.58	90.81/64.85	92.93/88.78	87.15	87.96	79.09
UNetMamba	92.54/86.12	96.54/94.63	85.96/75.39	86.37/76.01	95.47/91.32	90.39	91.37	84.41
TransUNet	93.08/87.06	96.88/93.94	86.74/76.59	87.66/78.03	96.40/93.05	91.03	92.15	85.73
UNetFormer	93.02/86.95	97.14/94.43	86.21/75.76	86.93/76.88	96.35/92.96	90.77	91.89	85.33
MAResU-Net	93.15/87.17	97.21/94.57	86.73/76.57	87.14/77.21	96.67/93.56	91.05	92.18	85.82
CMTFNet	93.08/87.06	97.30/94.73	87.13/77.20	86.89/76.97	90.22/82.18	90.97	91.89	85.28
RS3Mamba	92.95/86.12	97.32/94.79	85.97/75.39	86.60/76.37	96.44/93.13	90.77	91.89	85.24
DECSNet	92.35/85.78	97.07/94.31	85.43/74.57	86.33/75.94	95.24/90.92	90.29	91.29	84.31
MIFNet	92.97/86.86	97.23/94.60	86.74/76.58	87.23/77.35	96.91/94.01	91.01	92.07	85.81
DFENet	93.27/87.44	97.31/95.83	86.41/76.07	87.35/77.53	96.49/93.22	91.08	92.21	85.89

**Table 3:** Model complexity analysis and performance comparison on the ISPRS Vaihingen dataset.

Methods	FLOPs(G)	Param(M)	mIoU (%)
SFFNet	12.99	34.18	73.85
UNetMamba	4.34	13.89	80.42
TransUNet	38.57	105.32	82.04
UNetFormer	2.94	11.69	82.44
MAResU-Net	7.19	26.28	82.51
CMTFNet	8.87	30.07	82.52
RS3Mamba	9.87	43.32	82.56
DECSNet	10.07	47.41	81.79
MIFNet	8.84	25.36	82.71
DFENet	16.56	95.05	83.09

**Table 4:** Ablation experiment on the structure of DFENet. The best values are shown in bold.

Local Path	Global Path	mIoU (%)
✓		81.97
	✓	78.79
✓	✓	**83.09**

**Table 5:** Ablation experiment on the global path. The best values are shown in bold.

PAB	MAAB	SCAB	FFEB	mIoU (%)
	✓	✓	✓	78.31
✓		✓	✓	77.17
✓	✓		✓	76.42
✓	✓	✓		76.56
✓	✓	✓	✓	**78.79**

**Table 6:** Ablation experiment on FFEB. The best values are shown in bold.

FMU	HAU	mIoU (%)
✓		82.41
	✓	82.36
✓	✓	**83.09**

## Data Availability

The code will be made publicly available at https://github.com/LZS-HUB2000/DFENet, after the paper is accepted for publication, and can be accessed on 10 April 2026. The Vaihingen and the Potsdam datasets can be obtained from https://www.isprs.org/resources/datasets/benchmarks/UrbanSemLab/default.aspx (accessed on 12 February 2026).
